# Millet fermentations: a review on microorganisms, fermentation parameters, nutrients bio-utilization, and future prospects

**DOI:** 10.3389/fmicb.2026.1990322

**Published:** 2026-10-08

**Authors:** Yufeng He, Nai Li, Mingzhe Chen, Juan Bai, Ying Zhu, Jiayan Zhang, Yansheng Zhao, Yongjun Xia, Xiang Xiao

**Affiliations:** 1 School of Food Science and Engineering, Jiangsu University, Zhenjiang, China; 2 School of Agriculture and Biology, Shanghai Jiao Tong University, Shanghai, China

**Keywords:** artificial intelligence, bio-utilization, fermentation, millet, omics integration, online detection

## Abstract

Millets are prominent cereal materials worldwide. Fermentation is a typical bioprocessing method of millets, which boosts the sensory properties, nutritional composition and biological activity of millet products. In recent years, millets fermentation has been receiving increasing attention and many cutting-edge technologies have demonstrated significant application potential. This review provides an overview of recent progress in millets fermentation. The fermentation pattern, fermentation microorganisms, as well as process parameters are recognized as key factors that affect the fermentation process and product quality. Meanwhile, fermentation improves the bio-utilization of millet nutrients from aspects such as reducing starch digestibility, promoting the release and transformation of polyphenols, and increasing the bioaccessibility of minerals. Application of omics integration strategies, artificial intelligence models and online detection techniques exhibits great potential in exploring the fermentation mechanism and enhancing the fermentation efficiency and stability. These advancements are poised to significantly enhance industrial fermentation practices and highlights the value of fermented millets in healthy diets.

## 1 Introduction

Millets are coarse cereals belonging to the grass family “Poaceae.” The cultivation of millets was initially observed in northern China around 10,000 years ago (Lu et al., 2009). The main planting areas of millets include Africa, Southern Asia and Eastern Asia. India and China are the main growing and consuming countries of millets (Yameogo et al., 2022; Yadav et al., 2024). Globally, the most commonly grown millet varieties include pearl millet, barnyard millet, proso millet, finger millet, foxtail millet, sorghum, Job's tears, teff, fonio, browntop millet, little millet and kodo millet (Anitha et al., 2024). As C4 plants species, millets exhibit superior ability of converting carbon dioxide from the atmosphere into carbohydrates and higher nitrogen and water usage efficiencies (Peng and Zhang, 2021). Due to its strong tolerance of climate change, pests, and diseases, the significance of millets for global food security is becoming increasingly prominent.

Millets are a rich source of nutrients, which consists of approximately 55–73% carbohydrate (including 2%−9% dietary fiber), 6%−15% protein and 2%−8% fat. In addition to these macronutrients, millets also provide a variety of micronutrients, particularly iron, zinc, potassium, magnesium and calcium (Sheethal et al., 2022; Anitha et al., 2024; Kumar et al., 2024). The nutritional composition of different millet varieties also exhibits significant differences. For example, foxtail millet contained higher amounts of proteins (12 g/100 g) than barnyard millet (6 g/100 g) and finger millet (7 g/100 g) (Sharma et al., 2021). Compared to rice and wheat, millets are known to contain a good amount of dietary fibers and polyphenols (Aalim et al., 2025; Basharat et al., 2025). These bioactive components enable millet to control body weight, maintain gut health and support the body's defenses against oxidative stress (Mushtaq et al., 2021). It is important to note that millets contain also antinutrients such as phytic acid and tannins (Hemanth et al., 2025; Singh et al., 2025). These antinutritional components can bind with other dietary elements and hinder the digestion and absorption of certain nutrients (for example protein and minerals) (Hao et al., 2025). Due to the high content of dietary fiber and polyphenols, millet often falls short of wheat and rice in terms of taste and flavor (Jin et al., 2023). Therefore, the key issue that needs to be addressed in the processing of millet is how to improve the sensory quality of millet while retaining its specific nutritional components.

Fermentation is a cereal processing technique of great antiquity. The nature of fermentation is the utilization and transformation of nutrients by microorganisms (Wu et al., 2019). The traditional fermented products of millet are over the world, typical examples include *Fagao* from China, *Dosa* from India, and *Kunun-Zaki* from Nigeria (Adebiyi et al., 2018; Tomar et al., 2025; Wuni et al., 2025). These fermented foods are mainly produced through the spontaneous fermentation of microbial communities. In recent years, inoculated fermentation has been increasingly used to produce industrialized millet products such as millet beverage, millet porridge and millet flour (Wang et al., 2019b; Kumari et al., 2022). Compared with other processing techniques, fermentation not only enables the retention and removal of specific nutrient components, but also allows for the synthesis of new substances (Zhao et al., 2024b; He et al., 2025). In addition to its nutritional peculiarity and sensory quality, fermentation can significantly enhance the digestive properties and nutrient utilization of millet. For example, fermentation resulted in significant reductions of antinutrients levels in flatbread (Yehuala et al., 2025a). Another study indicated that the protein digestibility of foxtail millet protein was significantly improved by fermentation (Bai et al., 2025). However, there are still some limitations in the research on millet fermentation. For many bacterial strains, the metabolic mechanisms for millet components remain unclear. Besides, a large amount of fermentation data has not been effectively mined, which leads to insufficient precision during production and low efficiency.

In recent years, many cutting-edge technologies have been gradually applied to the fermentation of grains (Duan et al., 2026). By the combined application of technologies such as genomics, transcriptomics, metabolomics, and proteomics, the relationship among microorganisms, metabolic pathways and metabolites during the fermentation can be effectively identified (Li et al., 2025b; Wang et al., 2026). Based on artificial intelligence technology, data generated during fermentation can be used to establish neural networks, thereby making the fermentation process more precise and efficient (El-Mesery et al., 2024; Li et al., 2025a). By utilizing the online detection technology based on spectroscopy, it is possible to conduct real-time analysis of the key indicators of the fermentation process (Guo et al., 2020; Chen et al., 2023). Thereby, the contamination risks and data lag problems caused by sampling can be avoided.

This review provides an overview of recent progress in millets fermentation. It covers fermentation patterns, fermentation microbes and fermentation parameters of millet. Although existing reviews have mainly examined the nutritive peculiarity, the sensory qualities and the health benefits of fermented millets, this review focuses on the influence of fermentation on the digestive characteristics and nutrient utilization of millets. It also highlights the potential application values of omics integration, artificial intelligence technology and online detection technology in millets fermentation. The present review provided theoretical references for the utilization of fermented millets in functional cereal products.

## 2 Literature search strategy

This narrative review was conducted to provide a updated overview of millet fermentation, particularly in relation to fermentation process, millet nutrient digestion and utilization, and potential application of emerging technologies. To comprehensively examine these topics, a structured methodology was used for searching and integrating relevant reports. Academic databases and publisher platforms such as Web of Science, PubMed, ScienceDirect, Wiley and Chaoxing Discovery were used to identify the relevant literatures. To make sure a broad yet accurate coverage of these topics, multiple keywords were used, including combinations of terms such as “millet,” “fermentation,” “lactic acid bacteria,” “yeast,” “fermentation parameter,” “digestibility,” “bioaccessibility,” “bioavailability,” “omics integration,” “artificial intelligence” and “online detection.”

Literatures published within the past 5 years were predominantly considered, reflecting the latest progress in fermentation technologies and nutrients utilization. The selection of the literatures depended on several factors, including its relevance to the scope of the review, the journal JCR partition and impact factor, the rationality of the experimental design, and the sufficiency of experimental data. Non-English literatures, articles outside the research scope, articles without accessible full text, conference abstracts, preprints and non-peer-reviewed opinion pieces were excluded.

## 3 Fermentation patterns of millets

Based on the differences in fermentation microorganisms and water content, the fermentation patterns of millets can be further classified. This review elaborates on the main fermentation patterns of millets from two perspectives: spontaneous fermentation and inoculated fermentation, as well as liquid-state fermentation and solid-state fermentation (Figure 1). The following part will further discuss the differences of these fermentation patterns and their impact on fermentation technology and product quality.

**Figure 1:**
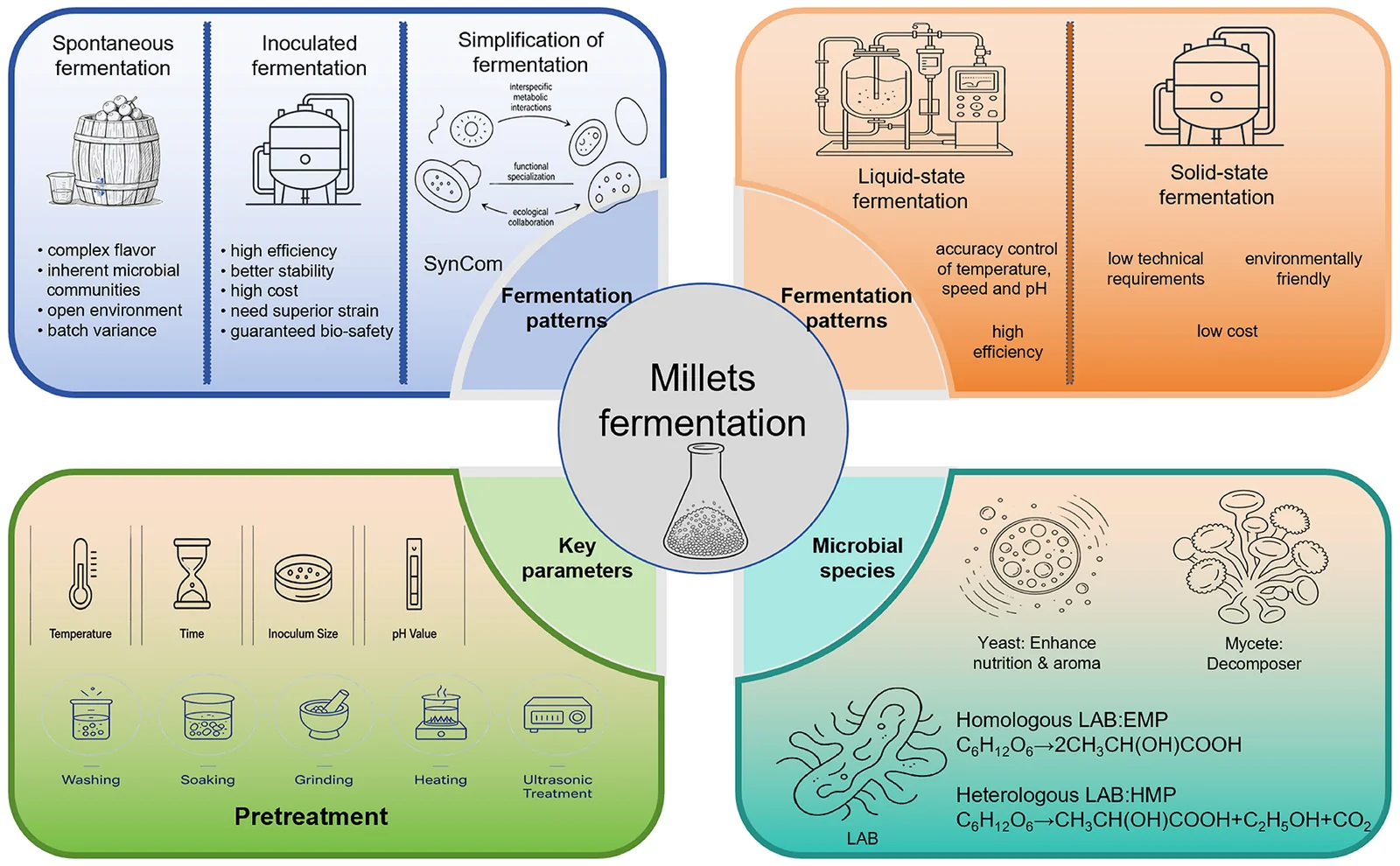
Fermentation patterns, fermentation microbes and fermentation parameters of millet. SynCom, synthetic microbial community; LAB, lactic acid bacteria.

### 3.1 Spontaneous and inoculated fermentation

#### 3.1.1 Spontaneous fermentation

According to whether a clearly defined starter culture is used during the fermentation process, the fermentation of millet can be further classified as spontaneous and inoculated fermentation (Navarrete-Bolaños, 2012). Spontaneous fermentation is a long-standing cereal processing method. It relies on the inherent microbial communities present in the raw materials and the environment to complete (Xia et al., 2019; Balcázar-Zumaeta et al., 2023). Millet products made through spontaneous fermentation processing include dough, porridge, vinegar and flour (Chen et al., 2016; Akinola and Osundahunsi, 2017; Mudau et al., 2022; Atter et al., 2025). During the spontaneous fermentation process, the microbial community exhibited dynamic characteristics. For example, during the early stage of the glutinous proso millet fermentation, the microbial community is dominated by *Pantoea* and *Lactobacillus*. However, in the later stage of fermentation, the microbial community is dominated by *Lactococus* (Bian et al., 2022). Spontaneous fermentation does not require sterilization of the raw materials. It usually involves steps such as soaking, cleaning, and grinding (Khazi et al., 2024; Atuna et al., 2025). It should be noted that, for spontaneous fermentation, the consistency across different batches of maybe relatively poor (Aldrete-Tapia et al., 2018). This can be attributed to the structure of inherent microbial community may vary between different batches. Because spontaneous fermentation is usually carried out in an open environment, it may be affected environmental factors such as temperature, humidity and spoilage organisms (Valentini et al., 2025; Yuan et al., 2026). Therefore, without proper control, spontaneous fermentation poses a relatively high risk to biological safety. Although thermal treatment can effectively kill pathogenic bacteria, heat-resistant mycotoxins (such as deoxynivalenol, aflatoxin and ochratoxin) cannot be effectively eliminated. It is worth noting that the period of spontaneous fermentation can be shortened by inoculation through back-slopping, which refers to using a small quantity of previously fermented raw materials in the new batch (Kimaryo et al., 2000; Reale et al., 2026). These added materials contain core microbial communities required for fermentation and can be used as starters (Madilo et al., 2024). In the production of *Ogi* (a traditional food made of maize and millet), back-slopping fermentation products exhibits a faster fermentation rate and a lower count of pathogenic organisms than spontaneous fermentation products (Teniola et al., 2005). Although back-slopping fermentation relies on starters, its microbial community is still unstable and uncontrollable. Therefore, back-slopping fermentation can still be regarded essentially as a form of spontaneous fermentation.

#### 3.1.2 Inoculated fermentation

Inoculated fermentation is accomplished by using specific strain or a combination of strains. The genetic background and metabolic potential of these microorganisms are clear. In order to ensure the stability of the fermentation process and prevent the fermentation strains from being contaminated, inoculated fermentation usually requires sterilization of the raw materials and relies on specialized fermentation equipment for operation (Xu et al., 2024). Compare with spontaneous fermentation, inoculated fermentation has better bio-safety and batch stability. In recent years, inoculated fermentation has been applied to the production of various products such as millet porridge, millet flour, and millet dough (Banwo et al., 2021; Chauhan et al., 2024; Lubaale and Duodu, 2024). Most of the strains used in inoculated fermentation are obtained through screening. Therefore, these strains usually possess excellent characteristics in certain aspects, such as rapid growth rate, strong environmental tolerance, and high bioconversion efficiency (Liu et al., 2025c; Sui et al., 2026). With the help of these strains, the fermentation period can be effectively shortened. During the spontaneous fermentation of millet flatbread, the total titrable acidity significantly increased after 144 hours of fermentation (Yehuala et al., 2025a). While in inoculated fermentation of millet dough, it only takes 48 h to achieve similar increment (Banwo et al., 2021). Due to its high efficiency and stability, inoculated fermentation has become the mainstream technology in industrial production.

#### 3.1.3 Simplification of spontaneous fermentation by inoculated fermentation

Although inoculated fermentation has become the mainstream production pattern, spontaneous fermentation still has its unique advantages. Many flavor-based fermented products still rely on spontaneous fermentation for production. The complex aroma substances of these products require the collaborative fermentation by multiple microorganisms. For example, during the fermentation of millet vinegar, *Acetobacter, Saccharomyces, Wallemia* and *Alternaria* was observed to be positively correlated with the production of certain amino acids and acetic acid, while *Lactobacillus, Staphylococcus* and *Massilia* were found to contribute positively to the production of oxalic acid and lactic acid (Li et al., 2026). This kind of complex bio-transformation is difficult to mimic through conventional inoculated fermentation. It is worth noting that the emergence of synthetic microbial community (SynCom) may change this situation. SynCom refers to an artificial ecosystem composed of two or more identified microbial strains, assembled under controlled conditions (Zhao et al., 2026). SynCom can exhibit predictable functions through interspecific metabolic interactions, functional specialization, and ecological collaboration (Meng et al., 2025; Zhang et al., 2026). By identifying and separating the species that play the core role in the spontaneous fermentation process, a SynCom can be initially established. Based on the microbial community analysis of spontaneously fermented sausages, *Lactobacillus, Staphylococcus, Macrococcus, Streptococcus*, and *Pediococcus* was identified as core genera. These core genera were further used to construct a SynCom. Sausages inoculated with this SynCom exhibited better quality of aroma profile than the samples inoculated with a commercial starter culture (Wu et al., 2023). Although SynCom have not been applied in millet fermentation, it provided feasible approach for simplifying and stabilizing the complex fermentation process.

### 3.2 Liquid-state fermentation and solid-state fermentation

#### 3.2.1 Liquid-state fermentation

According to the ratio of raw materials to water in the fermentation system, millet fermentation can be divided into two forms: solid-state fermentation and liquid-state fermentation. Millet products produced via liquid-state fermentation include millet milk, millet porridge and millet flour (Songré-Ouattara et al., 2009; Wang et al., 2019b; Gupta and Gaur, 2024). The solid-to-liquid ratio of the liquid-fermentation of millet is usually between 1:2 and 1:10. Liquid-state fermentation has the advantages of short fermentation time, high mass transfer efficiency and preferable producing repeatability. According to the existing researches, the duration of the liquid-state fermentation of millet were usually limited to within 72 h (Chen et al., 2022; Kumari et al., 2022; Samtiya et al., 2024). In the liquid-state fermentation environment, microorganisms can grow rapidly and consume nutrients within a short period of time. Therefore, long fermentation time can lead to problems such as insufficient nutrition and product inhibition, thereby reducing the efficiency of the fermentation (Jatoi et al., 2023). Liquid-state fermentation requires the use of specialized fermentation equipment. These equipments can achieve real-time monitoring and precise control of temperature, speed and pH, thereby ensuring the repeatability of the fermentation. By employing ventilation and feeding strategies, certain equipment can enable the continuous and efficient operation of liquid-state fermentation (Wang et al., 2019a). Through liquid-state fermentation, the nutritional composition, physical and chemical properties, and biological activity of millet can be effectively improved. For example, the soluble dietary fiber content of millet bran can be increased to 12% after fermentation, and the fermented millet bran exhibited significant effect in relieving diarrhea (Chen et al., 2022). Another study indicated that the water absorption capacity, and gel consistency of millet flour can be improved by fermentation (Paliwal et al., 2022).

#### 3.2.2 Solid-state fermentation

Solid-state fermentation is another common method for millet fermentation. For solid-state fermentation, the solid-to-liquid ratio is usually less than 1:2 (Venkateswaran and Vijayalakshmi, 2010; Purewal et al., 2020). In this situation, the materials are damp but cannot be completely covered by water. Therefore, the distribution of temperature, moisture and pH is likely to be uneven, and this may lead to the inconsistency of microbial growth and metabolic activity. Additionally, due to high solid-to-liquid ratio, the mass transfer efficiency of solid-state fermentation is lower than liquid-state fermentation. Based on the existing researches, the duration of the solid-state fermentation of millet were usually between 3 to 10 days (Venkateswaran and Vijayalakshmi, 2010; Gabriele et al., 2024). Because of the specific solid-to-liquid ratio, the solid matrix can provide physical support for the growth of fungal mycelia. Therefore, molds are commonly used microorganisms in the solid-state fermentation of millet. For fermentation not using mold, if the raw materials are not sterilized, the risk of mold contamination will increase significantly as the fermentation progresses. Millet products produced via solid-state fermentation include mold culture, millet dough and millet bread (Adepehin, 2020; Wang et al., 2023a; Endalew et al., 2024). Apart from the change in nutritional composition, the aroma quality of millet can also be improved after solid-state fermentation. During the primary fermentation stage of broomcorn millet *Huangjiu*, flavor compounds such as esters, alcohols, acids, aldehydes, ketones and phenols were identified. 5-methyl-2-(1-methylethyl)-1-Hexanol was considered as the key aroma substance that most closely related to the bacterial community (Wang et al., 2023a). Compared with liquid-state fermentation, solid-state fermentation does not require processes such as centrifugation and filtration for product collection. Another advantage of solid-state fermentation is that it generates less waste and requires less energy consumption (Thomas et al., 2013; Lizardi-Jiménez and Hernández-Martínez, 2017). Therefore, solid-state fermentation is more in line with the concept of green production.

## 4 Key parameters of millets fermentation

Fermentation parameters strongly affect both the success of millet fermentation and the quality of the resulting products. Key parameters of millet fermentation include temperature, time, inoculation dosage, pH and pretreatment method. A thorough understanding and necessary optimization of these parameters can ensure the efficient operation of the fermentation process. Fermentation parameters applied in millet fermentation were summarized in Table 1.

**Table 1:** Microorganisms and fermentation parameters applied in millet fermentation.

Products	Fermentation pattern	Microorganisms	Fermentation parameters	Refs
Millet flour	Spontaneous liquid-state fermentation	*Lactobacillus, Lactococcus, Pediococcus* spp.	37°C; 14 h; pH 3.9; Solid-liquid ratio: 1:3 (w/v)	Alkaltham et al., 2024
Broomcorn Millet *Huangjiu*	Spontaneous fermentation	*Pediococcus, Pantoea, Weissella, Saccharomyces* and *Rhizopus* spp.	Primary fermentation: 30°C, 5-8 d; Post-fermentation: 13-17°C, 30 d; Fortified qu addition dosage: 16%	Wang et al., 2023a
Millet vinegar	Spontaneous liquid-state fermentation	*Lactobacillus, Acetobacter* and *Aspergillus* spp.	38-45°C; 52 d; Moisture content: 650 g/kg cupei	Yan et al., 2025
Millet *Huangjiu*	Spontaneous fermentation	*Bacillus, Weissella, Paenibacillus, Klebsiella, Prevotella* spp.	28°C; 12 d	Yan et al., 2022
Finger millet based *Injera*	Spontaneous liquid-state fermentation	/	22°C; 96 h; Solid-liquid ratio: 1:3 (w/v)	Endalew et al., 2024
Millet sourdough	Spontaneous solid-state fermentation	*Bacillus, Brevibacillus, Acinetobacter* and *Pediococcuss* pp.	28°C; 48 h; pH from 6 to 4 Solid-liquid ratio: 2:1 (w/v)	Adepehin, 2020
Millet flour	Spontaneous liquid-state fermentation	*/*	30°C; 24 h; pH from 6.6 to 5.1 Solid-liquid ratio: 1:8 (w/v)	Gupta and Gaur, 2024
Millet *Idli* batter	Spontaneous liquid-state fermentation	Mainly lactic acid bacteria spp.	30°C; 24 h; pH from 6.2 to 3.0 Solid-liquid ratio: 1:2 (w/v)	Chelliah et al., 2017
Pearl millet dough	Spontaneous fermentation	Mainly lactic acid bacteria and yeast spp.	22°C; 7 d; Solid-liquid ratio: 1:1 and 1:3 (w/v)	Yehuala et al., 2025a
Finger millet flour	Inoculated solid-state fermentation	*Monascus purpureus*	28°C; 10 d; pH from 5.1 to 3.8 Moisture content: 32%; Inoculation dosage: 2% spore suspension	Venkateswaran and Vijayalakshmi, 2010
Fermented millet grain	Inoculated solid-state fermentation	*Aspergillus oryzae* MTCC 3170 *Rhizopus azygosporus* MTCC 110195	28°C; 336 h; Solid-liquid ratio: 10:1 (w/v) Inoculation dosage: 1 × 10^4^ spores/g sample	Purewal et al., 2020
Pearl millet grain	Inoculated solid-state fermentation	*Aspergillus sojae* MTCC 8779 *Rhizopus azygosporus* MTCC 10179	25°C; 7 d; Solid-liquid ratio: 10:1 (w/v)	Purewal et al., 2024
Pearl millet flour	Inoculated liquid-state fermentation	*Saccharomyces cerevisiae* ATCC 24860	38-45°C; 52 d; Moisture content: 650 g/kg cupei	Wu et al., 2006
Pearl millet flour	Inoculated liquid-state fermentation	*Saccharomyces cerevisiae*	30°C; 18 h; Solid-liquid ratio: 2:7 (w/v) Inoculation dosage: 1 × 10^9^ CFU/g sample	Kumari et al., 2022
Millet flour	Inoculated liquid-state fermentation	*Pediococcus acidilactici*	37°C; 48 h; Solid-liquid ratio: 1:3 (w/v) Inoculation dosage: 10^8^ CFU/mL sample	Zhang et al., 2025
Kodo millet flour	Inoculated fermentation	*Lactobacillus acidophilus* ATCC 4356	37°C; 72 h; pH 4.5 Inoculation dosage: 10^6^ CFU/mL sample	Dash et al., 2025
Finger millet flour	Inoculated liquid-state fermentation	*Lactobacillus salivarius* LMG 9477	30°C; 48 h; Solid-liquid ratio: 1:10 (w/v) Inoculation dosage: 10^6^ CFU/g sample	Mbithi-Mwikya et al., 2000
Dehulled millet	Inoculated solid-state fermentation	*Lactiplantibacillus plantarum*	30°C; 7 d; pH from 6.5 to 5.0 Solid-liquid ratio: 1000:1 (w/v) Inoculation dosage: 10^4^ CFU/g sample	Chauhan et al., 2024
Fermented millet flour	Inoculated liquid-state fermentation	*Lactiplantibacillus plantarum* F2	30°C; 48 h; pH 3.5 Solid-liquid ratio: 1:16.7 (w/v) Inoculation dosage: 4.5 × 10^6^ CFU/mL sample	Lubaale and Duodu, 2024
Foxtail millet grain	Inoculated liquid-state fermentation	*Lactiplantibacillus plantarum* CGMCC 14177	37°C; 72 h; pH from 7.0 to 3.6 Solid-liquid ratio: 1:2 (w/v) Inoculation dosage: 10^6^ CFU/mL sample	Chang et al., 2024
Millet bran	Inoculated liquid-state fermentation	*Bacillus subtilis* CICC 10453	36°C; 47 h; pH from 7.0 to 3.6 Solid-liquid ratio: 1:10 (w/v) Inoculation dosage: 10^7^ CFU/mL sample	Chen et al., 2022
Pearl millet flour	Inoculated liquid-state fermentation	*Limosilactobacillus fermentum* MS005 *Lactobacillus rhamnosus* GG	37°C; 24 h; pH from 6.3 to 3.9 Solid-liquid ratio: 1:4 (w/v); Inoculation dosage: 10^8^ CFU/mL sample	Samtiya et al., 2024
Broomcorn millet sour porridge	Inoculated liquid-state fermentation	*Lactobacillus brevis* L1 *Acetobacter aceti* A1 *Saccharomyces cerevisiae* E4	30°C; 32 h; Solid-liquid ratio: 1:3.3 (w/v) Inoculation dosage: 10^10^ CFU/mL sample	Wang et al., 2019b
Pearl millet flour	Inoculated liquid-state fermentation	*Lactobacillus sanfranciscensis Lactobacillus pentosus*	38°C; 96 h; pH 4.0 Solid-liquid ratio: 2:3 (w/v)	Gabriele et al., 2024

### 4.1 Temperature

Temperature can affect the growth and metabolic rate of microorganisms. Essentially, it regulates the enzymatic activities of microorganisms. An increase in temperature within the appropriate range can accelerate metabolic reactions. But beyond the optimal range, it will result in enzyme inactivation and then metabolic inhibition (Qian et al., 2023). The temperature differs between different patterns of millet fermentation. For example, the temperature of millet spontaneous fermentation (usually between 20–30°C) is usually lower than that of inoculated fermentation (usually between 30–40°C) (Endalew et al., 2024; Yehuala et al., 2025b). Notably, the fermentation temperature of the mold culture is significantly higher than that of other spontaneous fermentation processes, which can reach 45–50°C (Wang et al., 2023a). The high-temperature environment can selectively promote the growth of microorganisms such as *Bacillus* spp. and *Thermoactinomyces vulgaris* (Yang et al., 2009; Zhao et al., 2024a). These microorganisms encode a rich array of enzymes such as amylase and protease, which can facilitate the Maillard reaction and generate aroma precursor substances like pyrazines and furans (Liu et al., 2023). Furthermore, there are significant differences in the optimal fermentation temperatures for different types of microorganisms. Studies have shown that the fermentation temperatures of millet by molds were generally less than 30°C (Venkateswaran and Vijayalakshmi, 2010; Purewal et al., 2020). The temperature for yeast fermentation of millet is usually 25–30°C (Wu et al., 2006; Kumari et al., 2022). When LAB were used for millet fermentation, the optimal temperatures were usually between 30–38°C (Farooq et al., 2017; Dash et al., 2025). The fermentation temperature is also an important factor affecting the yield of the final products. During the fermentation process of *Huangjiu*, the ethanol yield showed a trend of increasing first and then decreasing as the fermentation temperature increased (Li et al., 2024b). In order to improve the final product yield, the temperature preferences of fermentation microorganisms and their key enzymes are crucial issues that need to be taken into consideration.

### 4.2 Time

Fermentation time is another key variable that regulates the fermentation process of millet. It strongly affects the microbial succession and nutritional composition in millet fermentation. During millet sourdough fermentation, the total bacteria count in the fermentation system increased rapidly within the first 12 h, and showed no significant change from 12 to 48 h. Different from total bacteria count, a significant reduction of LAB count was observed after 12 h of fermentation (Adepehin, 2020). Intra-species inhibitory effects such as quorum sensing and bacteriocins production may explain the changes of LAB. Another study investigated the microbial succession during the fermentation process of millet *Huangjiu* (Yan et al., 2022). As the fermentation time increases, the diversity of the bacterial community significantly decreases. *Bacillus* gradually became the most dominant genus. With these changes in the microbial communities, the content of reducing sugars in the fermentation system keeps increasing, while the levels of ethanol and acidity decreased. In addition to the microbial community changes, the content of polyphenols in millet also varies with the fermentation time (Purewal et al., 2020). As the fermentation progressed, the contents of water-soluble polyphenols and alcohol-soluble polyphenols in the millet continuously increase. When the fermentation time exceeds 240 h, this trend no longer existed. Similar results were also observed in the production of ethanol using millet as substrate (Wu et al., 2006). These evidences indicate that extending the fermentation time will not lead to a continuous increase in the yield of the final product. Optimizing the fermentation time is a necessary step to improve the efficiency of the millet fermentation.

### 4.3 Inoculation dosage

The inoculation dosage is one of the key parameters that regulates the initiation speed, metabolic efficiency, and product quality of fermentation. The essence of this parameter is the initial number of viable cells that inoculated to the fermentation system. In different millet inoculated fermentation studies, the initial numbers of viable cells in fermentation vessel ranged between 10^4^ CFU/g and 10^9^ CFU/g (Wu et al., 2006; Purewal et al., 2020; Kumari et al., 2022; Samtiya et al., 2024). Compared to bacteria and yeast, the inoculation dosages of mold were much lower. The initial numbers of spores were usually not exceeding 10^7^ CFU/g in millet fermentation. This is because the spores and hyphae of molds are large in size, and only a small amount of spores can ensure that the hyphae effectively cover the materials. Furthermore, a higher inoculation dosage can lead to the entanglement of the mycelium, thereby reducing the mass transfer efficiency (Chen et al., 2025). Studies have shown that the inoculation dosage exerted a non-linear effect on the fermentation of millet. When *Bacillus natto* was used in millet bran fermentation, increasing the inoculation dosage can effectively enhance the content of soluble dietary fiber (SDF) in millet bran. However, when the inoculation dosage exceeded 7%, the content of SDF in the fermentation product began to decrease (Chen et al., 2022). In another study, a multiple-strain starter culture was used in broomcorn millet sour porridge fermentation. The effect of the inoculation dosage on the sensory score of millet porridge increased firstly and then decreased (Wang et al., 2019b). Therefore, optimizing the inoculation dosage for millet fermentation is beneficial for improving product quality and avoiding excessive use of the start culture.

### 4.4 pH

Monitoring and controlling pH during millet fermentation is essential, as it influences enzymatic activity, microbial composition and product quality. The enzymatic breakdown of starch, proteins, and other macromolecules can be influenced by pH. For example, during the fermentation of millet by *Pediococcus acidilactici*, the pH of the fermentation product decreased from 6.45 to 3.16. At the same time, the content of amylose in the fermentation product significantly increased, while the size of the starch granules significantly decreased (Zhang et al., 2025). Acid hydrolysis might be an important reason of these effects. Relationships between pH changes and protein structure were also observed. After acid treatment, the average particle size of rice protein isolate was significantly reduced and this can enhance the creaming stability in the emulsion (Shen et al., 2023). In addition, pH exerts a complex effect on fermentation microorganisms. Fermenting microorganisms such as LAB and yeast can produce organic acids and grow rapidly in an acidic environment (pH 4–6) (Hu et al., 2021). This helps them to gain dominance within the microbial community and inhibit the growth of harmful microorganisms. On the other hand, an excessively low pH level will inhibit the further growth and the metabolic activity of these microorganisms (Adepehin, 2020). Therefore, maintaining a stable pH during the fermentation process enables microorganisms to maintain high metabolic activity for a relatively long period of time. Online pH detection and automatic feeding are the conventional methods to achieve this goal (Jiang et al., 2010). However, the acid-base neutralization reaction will produce a large amount of sodium salts, which will subsequently have a negative impact on the flavor of the product.

### 4.5 Pretreatment

Pretreatment of raw material is an important means to ensure the success of millet fermentation. In millet fermentation, the previously reported pretreatment methods include washing, soaking, grinding, heating and ultrasonic treatment. For unprocessed grains, washing is a necessary process to remove impurities and insects. Soaking can make the grain to become softer in texture and activate the endogenous enzymes. The soaking process is usually carried out at room temperature (18–26°C), with the duration ranging from a few h to one day (Chelliah et al., 2017; Purewal et al., 2020; Chauhan et al., 2024). Soaking also helps to promote the proliferation of the inherent microbial community of millet, thereby enhancing the efficiency of spontaneous fermentation. The main purpose of grinding is to increase the surface area of grains, so that microorganisms can have direct contact with a greater variety of nutrient molecules. After fermentation, the dehulled millet showed higher level of starch content than whole-milled millet (Onyango et al., 2023). This indicates that grinding promotes the utilization of starch by microorganisms. The heat treatment methods for before millet fermentation include hydrothermally treatment and steam treatment. It was reported that the resistant starch content decreased after steam treatment (100°C) (Onyango et al., 2023). A partial gelatinization of the starch can explain this reduction of resistant starch content (Ren et al., 2016). However, the gelatinization of starch in millet requires a relatively high temperature. Therefore, the decreased level of resistant starch was not observed in hydrothermally treatment (70°C) millet. Ultrasound technology has been explored as a promising method for improving the biological function of cereals. When ultrasonic treatment was applied at different temperatures (40°C and 60°C), the total phenolic content and total flavonoid content of millet significantly increased. This alteration of polyphenol content also led to enhanced antioxidant capacities (Alkaltham et al., 2024). Ultrasound treatment can cause damage to the cell walls of grains, thereby facilitating the release of free phenolic compounds during fermentation.

## 5 Microorganisms of millets fermentation

For both spontaneous and inoculated fermentation of millet, microorganisms play a crucial role in this process. Microorganisms that involved in millet fermentation include bacteria, yeast and mold. These microorganisms can enhance the quality of millet by degrading macromolecules, producing bioactive compounds and generating aroma compounds. The following part summarizes the changes in the microbial community during the spontaneous fermentation process of millet, as well as the fermentation characteristics of different types of microorganisms and their impacts on the quality of millet.

### 5.1 Microbial dynamics in spontaneous fermentation

During the spontaneous fermentation process of millet, the inherent microbial communities grow rapidly under suitable conditions. The dominant microbes gradually occupy the main ecological niches, ultimately endowing millet with unique flavor and nutritional peculiarity. Both bacteria and fungi are involved in this microbial community succession. For example, during the fermentation of broomcorn millet *Huangjiu*, both the bacteria and fungi community exhibited dynamic changes. In the initial stage of fermentation, the bacterial community was dominated by *Pantoea, Klebsiella, Weissella*, and the fungi community was dominated by *Rhizopus* and *Lichtheimia*. After 6 days of fermentation, the bacterial community was dominated by *Pediococcus*, and the fungi community was dominated by *Saccharomyces* (Wang et al., 2023a). For the same fermented millet food, due to differences in its production area, fermentation temperature, and crop variety, the microbial community will show significant differences in composition. Overall, the differences in the composition of fungi communities are greater than those in the composition of bacteria communities. Geography and millet variety rather than fermentation temperature played a fundamental role in microbial composition (Sun et al., 2023). This result highlights the significant role of the inherent community in the formation of the final microbial composition. Liu et al. investigated the relationship between microbial communities and flavor perception during millet bread fermentation. The results indicate that *Lactobacillus, Enterococcus, Pediococcus, Brevibacterium, Staphylococcus, Saccharomyces* were closely correlated with taste and aroma flavor compounds of the millet bread (Liu et al., 2025a). These results can provide a reference basis for the targeted development of millet fermentation starters.

### 5.2 Microbial species used in inoculated fermentation

#### 5.2.1 Bacteria

Bacterial strains that used for millet fermentation can be classified into LAB, acetic acid bacteria and bacillus. Among these microorganisms, LAB are the most commonly used species. LAB can efficiently utilize the carbohydrate in grains. Depending on the different metabolic products, they can be further classified into homofermentative LAB and heterofermentative LAB. Lactic acid is the main fermentation products of homologous LAB. For heterologous LAB, in addition to lactic acid, these strains also produce ethanol and carbon dioxide (Mubeen et al., 2025). Studies found that species such as *Ligilactobacillus salivarius* and *Lactiplantibacillus plantarum* can increase the total phenolic content and optimize the composition of amino acids of millet (Lubaale and Duodu, 2024; Mbithi-Mwikya et al., 2000). In addition to nutrients composition, the structure of macromolecules can also be changed by fermentation of LAB, leading to modifications of physicochemical properties. For example, after fermentation of *Lp. plantarum*, the millet starch exhibited enhanced proportion of short-branched chain, crystallinity, and short-range ordered degree. These structural alterations further improved the solubility, adsorption capacity, and pasting viscosity of starch (Chang et al., 2024). In another study, millet bran was fermented with *B. natto*, leading to better structural and functional properties of its dietary fiber (Chu et al., 2019). The fermentation capabilities of *Lactobacillus brevis* and *Acetobacter aceti* on millet sour porridge was compared in another study. Although the titratable acidity of the *A. aceti* fermented samples was significantly higher than that of the *L. brevis* fermented samples, these samples showed similar sensory scores (Wang et al., 2019b). Therefore, acetic acid bacteria can also be used in the production of fermented millet sour porridge.

#### 5.2.2 Yeast

Although the metabolic products of carbohydrates produced by heterologous LAB and yeast are similar, yeast exhibit higher efficiency in producing ethanol and carbon dioxide. Therefore, the application of yeast is more widespread in specific conditions such as dough fermentation and alcohol fermentation. The optimal fermentation temperature for yeast (25–30°C) is usually lower than that of LAB (30–38°C). Besides, yeast exhibits a better tolerance to high sugar environments. These characteristics can facilitate the yeast to occupy of the dominant ecological niche within the microbial community (Randez-Gil et al., 2013). Due to producing a large amount of carbon dioxide, yeast can facilitate the dough to form a porous and loose texture (Li et al., 2022). Yeast species that were used in millet fermentation include *Saccharomyces cerevisiae* and *S. boulardii* (Balli et al., 2023). Increased essential amino acids, phenolic acid and flavonoid content was observed when pearl millet flour was fermented by *S. cerevisiae* (Kumari et al., 2022). In addition to improving the nutritional quality, yeast can also enhance the aroma quality of millet. Through multiple metabolic pathways, yeast can convert precursor substances such as amino acids and fatty acids into aroma compounds such as alcohols, aldehydes and esters (Ma et al., 2022). In the fermentation of foxtail millet sourdough, product fermented with yeast contained high levels of 2, 6, 11-trimethyl dodecane, heptanal and octanoic acid ethyl ester, while these compounds were not enriched in product fermented with LAB strains (Li et al., 2024a). Therefore, yeast is particularly suitable for enhancing the aroma quality of fermented millet.

#### 5.2.3 Mold

Mold plays the role of “decomposer” during the fermentation process of millet. The main reason for this is that mold possess a unique extracellular enzyme secretion system and filamentous mycelial structure. The extracellular enzymes that mold use to break down macromolecules mainly include amylase, glucoamylase, protease, lipase (Wang et al., 2025a). Besides, the reticular mycelium can penetrate deep into the grain matrix, significantly increasing the contact area with the substrate. By continuously secreting enzymes, the grain macromolecules that near the tip of the mycelium can be effectively broken down. The molds used for the fermentation of millet include *Aspergillus sojae, A. oryzae, Rhizopus azygosporus* and *Monascus purpureu*. During pearl millet starch fermentation, both *R. azygosporus* and *A. sojae* can alter the structure of starch granules, leading to decreased particle size and increased solubility. Compared with *R. azygosporus, A. sojae* exerted a more significant impact on the structural and physical properties of millet starch (Purewal et al., 2024). Mold fermentation also had a significant impact on the protein content in millet, mainly manifested as a decrease in the total protein content and an increase in the soluble protein content (Venkateswaran and Vijayalakshmi, 2010). These studies further confirmed the role of mold as a “decomposer” in the fermentation of millet. Because LAB and yeast cannot efficiently utilize biological macromolecules, the presence of mold can provide sufficient substrates for the growth and metabolism of these microorganisms, thereby further endows fermented millet with its unique nutritional and flavor characteristics.

#### 5.2.4 Multiple strains inoculant

During fermentation, different microorganisms can form a complex network through dynamic interactions like cross-feeding and metabolic competition. These interactions can contribute to promoting the growth of specific microbes, maintaining the stability of the microbial community, and inhibiting the growth of spoilage microorganisms. For example, *Lp. plantarum* can produce organic acids to serve as the carbon source for the growth of *S. cerevisiae*, which then promoted the synergistic growth of each other (Sieuwerts et al., 2018). In recent years, an increasing number of mixed cultures were applied in the fermentation of millet. The reported strain combinations include the blending of different LAB strains, the blending of LAB and yeast, and the blending of LAB and acetic acid bacteria (Samtiya et al., 2024). In millet sour porridge fermentation, higher sensory evaluation score and titratable acidity was observed in multi-strain fermented product than single-strain fermented product (Wang et al., 2019b). This indicated that multi-strain fermentation can enable millet to have a greater sensory quality and stronger process stability. Another study indicated that co-cultures of the same genus may lead to lower fermentation efficiency than co-cultures of the different genera (Farooq et al., 2017). Therefore, how to legitimately design the combination of strains is the key issue for improving the fermentation efficiency and product quality. Using whole-genome sequencing technique, the metabolic potential of different strains can be further explored. By conducting tools such as genome-scale metabolic model (GSMM), the complementary metabolic potential of different strain combinations can be predicted (Liu et al., 2025b). For example, GSMM was used to predict the hydrogen production of 298,378 microbial co-culture combinations. The results showed that the combination of *Clostridium beijerinckii* and *Yokenella regensburgei* can achieve the highest hydrogen production, and this was further verified by the cultivation experiment (Schwalm et al., 2019).

## 6 Effect of fermentation on bio-utilization of millet nutrients

Millet is considered as a rich source of multiple nutrients. For different nutrients, the expectations for their bioavailability varies. Due to the increased incidence of metabolic diseases, it is necessary to reduce the total amount of absorbable carbohydrates and control their digestion and absorption (Wang et al., 2023b). For nutrients such as polyphenols and minerals, higher bio-utilization is needed. It is worth noting that fermentation can exert beneficial effect on bio-utilization of millet nutrients (Figure 2). The following part will elucidate how fermentation affect the bio-utilization of millet starch, polyphenols and minerals.

**Figure 2:**
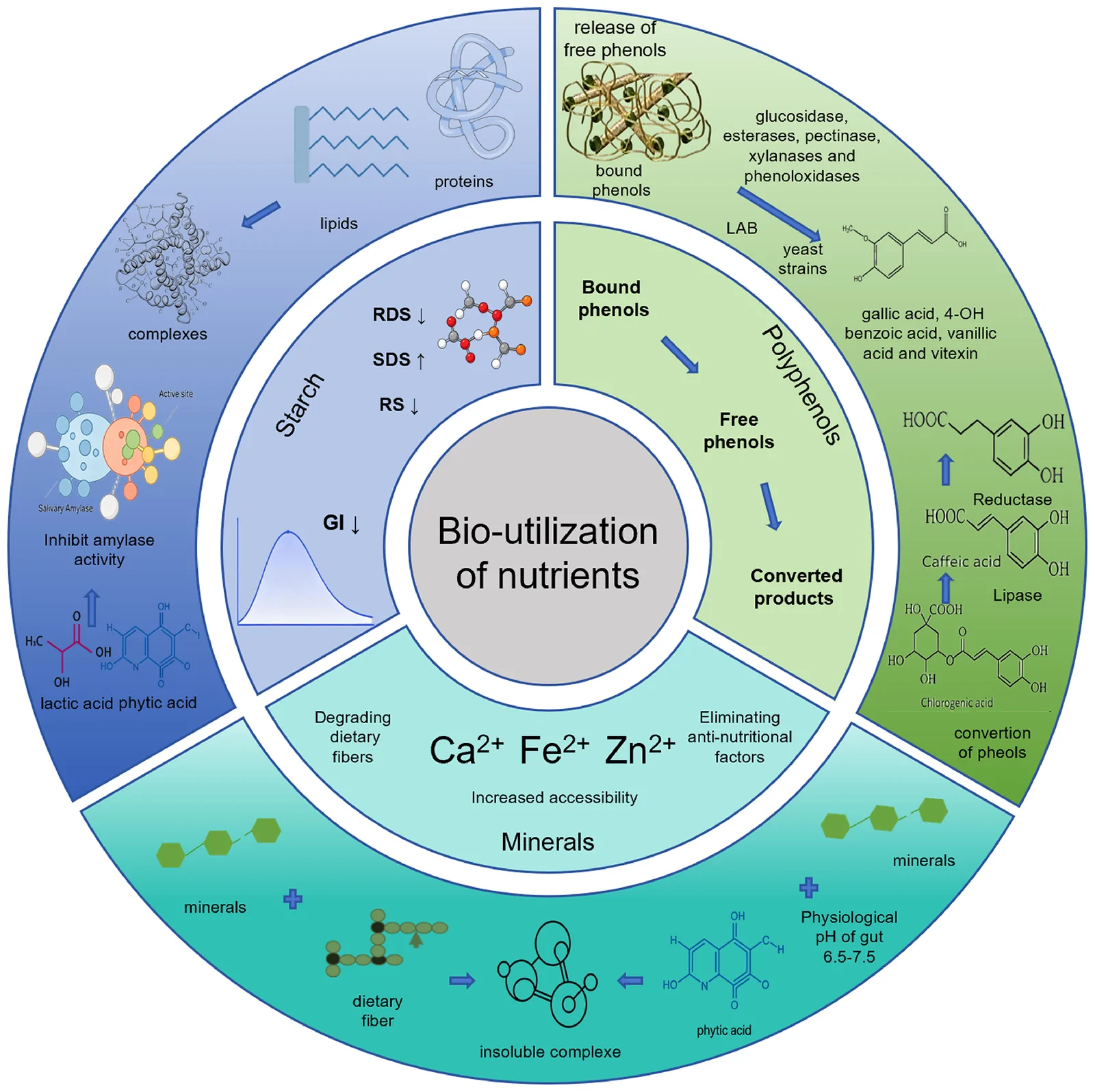
Fermentation affects the bio-utilization of millet nutrients. RDS, rapidly digestible starch; SDS, slowly digestible starch; RS, resistant starch; GI, glycemic index; LAB, lactic acid bacteria.

### 6.1 Starch digestion characteristics

In recent years, the incidence of diabetes has been continuously increasing worldwide (Wang et al., 2022b). The excessive intake of digestible carbohydrates is considered as the main cause of this trend. Starch contributes 67–79% of the millet whole grain, therefore the digestive characteristics of starch can exert direct impact on the glycemic response of millet (Chauhan et al., 2024). Sharma et al. investigated the effect of fermentation on the *in vitro* digestibility of millet starch. After 20 h of fermentation by LAB, the *in vitro* digestibility of millet starch was significantly enhanced. At the same time, fermentation also leads to a decrease in the content of anti-nutritional substances such as phytic acid, saponins and tannins, and this may explain why the digestion of starch was promoted (Sharma et al., 2022). However, another *in vitro* study reached a different result. Different varieties of millet were fermented by *Lp. plantarum* in an anaerobic environment for 7 days, and decreased predicted glycaemic indices were observed. Besides, fermentation reduces the proportion of rapidly digestible starch and increases the proportion of resistant starch (Chauhan et al., 2024). Regarding the influence of lactic acid bacteria fermentation on the digestion characteristics of millet starch, the above studies reached different conclusions. The difference in the analysis methods for digestion characteristics might be the main reason for this result. Therefore, similar studies need to adopt a unified *in vitro* digestion evaluation method (such as INFOGEST protocol). Besides, the differences in fermentation time may be another the reason for the different results. As the fermentation process progresses, the starch is gradually consumed by microorganisms, thus the content of rapidly digestible starch significantly decreases. Meanwhile, the content of lactic acid gradually increased, further exerting the inhibitory effect on amylase. Similar results were reported by Azeez et al., who carried out solid-state fermentation of brown millet using *S. cerevisiae* (Azeez et al., 2022). It was worth noting that biological macromolecules such as lipids and proteins also affect the digestive properties of starch in fermented millet. For example, lipids result in the formation of starch-lipid complexes, and further lead to slower digestion rate. The presence of proteins such as albumin, globulins and glutenins can form matrixes around the starch granules that acts as a barrier toward starch hydrolytic enzymes (Annor et al., 2017). Although a relatively well-established *in vitro* digestion protocol has been applied by many studies, it still has fundamental differences from the *in vivo* digestion and absorption processes (Rasera et al., 2023). Bionic digestive systems and *in vivo* models are recommended for obtaining more accurate research data.

### 6.2 Release and transformation of polyphenols

Phenolic compounds are important bioactive substances in millet, which can act as free radical scavenger and regulate action of enzymes involved in glucose and lipid metabolism (Wu et al., 2020; Chen and Wang, 2022). The phenolic compounds in millet can be classified into phenolic acids, flavonoids and phytosterols (Kumari et al., 2025). In millet, phenols are characterized in free forms and bound forms. Bound phenols are covalently linked to cytoderm materials such as structural proteins and fibers. These matrixes can not be easily absorbed and exhibits lower biological activity (Li et al., 2025c). Therefore, fermentation is an effective way to enhance the bio-accessibility of phenols in millet. The influence of fermentation on the polyphenols in millet mainly manifests in two aspects: the bound phenols are dissociated into free forms; the free phenols are further transformed into other metabolic products. For example, when pearl millet was fermented by LAB and yeast strains, levels of free form gallic acid, 4-OH benzoic acid, vanillic acid and vitexin significantly increased, indicating the release of free phenols. This process was accompanied by the enhanced *in vitro* antioxidant activities (Gabriele et al., 2024). Microbial enzymes that involved in the release of free phenols include glucosidase, esterases, pectinase, xylanases and phenoloxidases (Zhou et al., 2020). During the fermentation process of millet vinegar, the content of vanillic acid, caffeic acid, vitexin increased firstly and then decreased. This indicates that these phenolic compounds may first be released, and then be converted into other metabolites by certain microbes. The correlation analysis indicated that *Aspergillus ruber* and *Lactobacillus* spp. may involve this biotransformation process (Yan et al., 2025). Studies have shown that the microbial transformation products of epicatechin and anthocyanin exhibited stronger antioxidant activity (Bao et al., 2019; Chen et al., 2020). However, the microbial transformation mechanism of phenolic compounds during the fermentation process of millet has not yet been thoroughly studied. For analyzing the bio-transformation of phenols, the isotopic tracer technique may be considered as an effective method. Besides, using transcriptomics techniques, the metabolic mechanism of phenols by microorganisms can be further analyzed.

### 6.3 Bioaccessibility of minerals

In comparison to wheat, rice, or maize, millet exhibits a higher ash content (up to 3%) (Krishnan and Meera, 2018; Dash et al., 2025). However, the presence of phytic acid, phenols and dietary fibers can reduce the bioaccessibility and bioavailability of minerals. For example, phytic acid forms complex with minerals and the complex becomes insoluble at the physiological pH of the gut. Based on the results of a clinical trial, phytic acid exerted a dose-dependent inhibiting effect on the absorption of iron, zinc and calcium (Brune et al., 1992). Endalew et al. reported that increased levels of bioaccessible iron, calcium and zinc were observed in millet based *Injera* after spontaneous fermentation, and this was accompanied by the decreased level of phytic acid (Endalew et al., 2024). This indicated that fermentation can enhance the bioavailability of minerals by reducing the content of antinutritional factors. It was indicated that phytate can be converted into short-chain fatty acids though microbial cross-feeding between *Mitsuokella jalaludinii* and *Anaerostipes rhamnosivorans* (De Vos et al., 2024). Besides, certain microbes can produce organic acids such as citric, malic, or lactic acid. These compounds can reduce the pH value of the fermentation system and form soluble ligands, thereby enhance the activity of phytase (Teucher et al., 2004). Because minerals can form insoluble complexes with dietary fibers, the moderate degradation of dietary fiber also helps to enhance the bioavailability of minerals. It was reported that *B. natto* fermentation promoted the degradation of cellulose and hemicellulose, and increased the soluble dietary fiber content of millet bran (Chu et al., 2019). Therefore, fermentation can enhance the bioavailability of minerals by eliminating anti-nutritional factors and degrading dietary fibers.

## 7 Potential application of emerging technologies in millet fermentation

The existing research on millet fermentation mostly relies on traditional methods. By applying emerging techniques such as multi-omics integration and artificial intelligence tools, some complex scientific issues of millet fermentation are expected to be further resolved. Besides, the application of online detection technology in millet fermentation will contribute to improve the production efficiency and maintain the product quality and safety (Figure 3).

**Figure 3:**
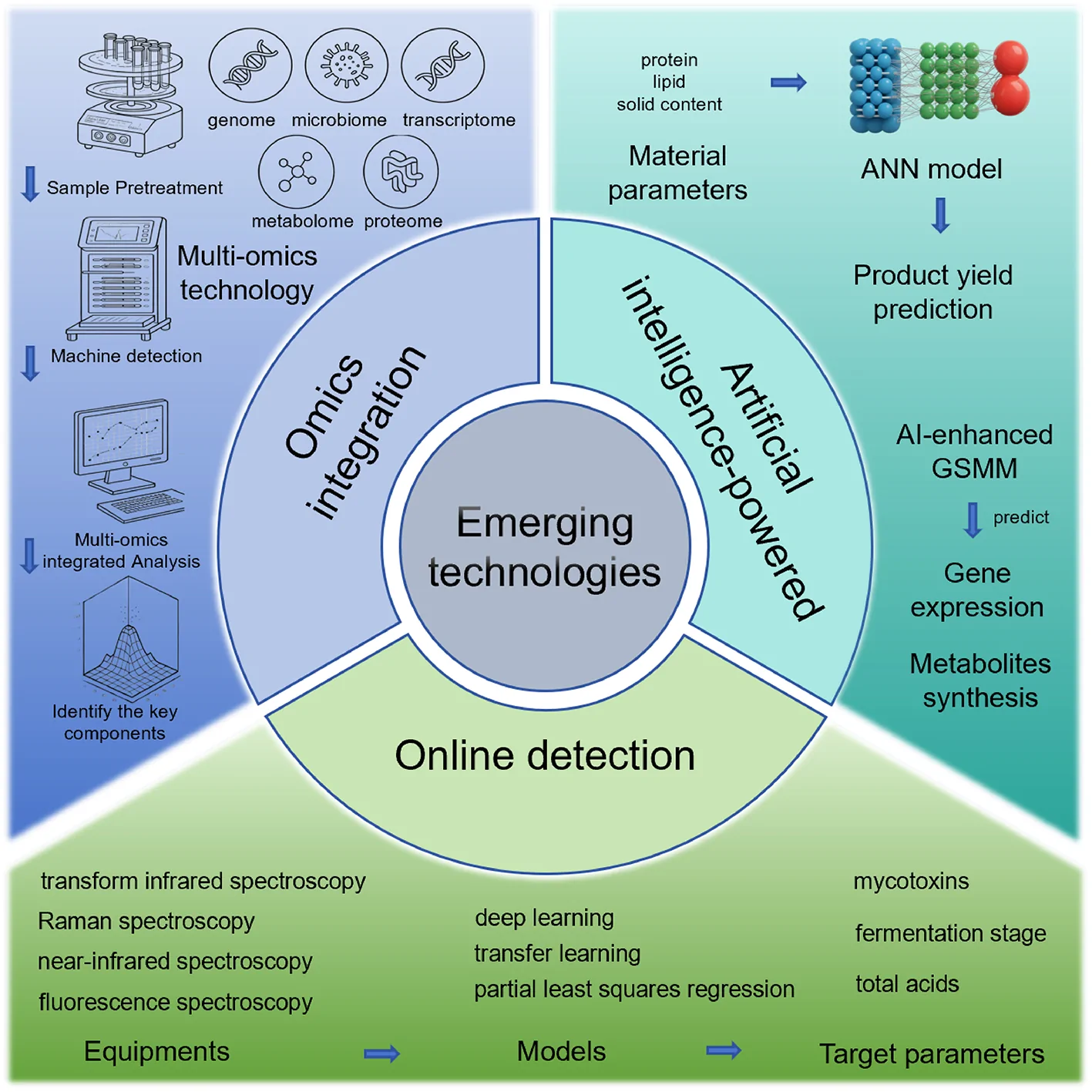
Emerging techniques applied in millet fermentation. ANN, artificial neural network; GSMM, genome-scale metabolic model.

### 7.1 Omics integration strategies

Omics integration refers to the systematic analysis of single-omics data. The data levels covered by the multi-omics research include the genome, microbiome, transcriptome, metabolome and proteome (Cassotta et al., 2021; Wen et al., 2024). In millet fermentation, there often exists a complex correspondence between multiple microbial species and different metabolic products. The conventional methods are unable to grasp the entire picture of the microbial strains and metabolites, and the key roles within them also cannot be effectively identified. Remarkably, multi-omics technology can explain the fermentation process from the perspectives of microbial species-genetic background-metabolic pathways-metabolic products. Yan et al. used high throughput sequencing and metabolomics to analyze the potential relationship between the microbial community and polyphenols. Based on the results of the correlation analysis, significant positive correlations between *Lactobacillus harbinensis* and caffeic acid, kaempferic acid, kaempferolide levels were observed. Using whole-genome sequencing technique, carbohydrate hydrolases, glycosidases and cellulases were further identified. These multi-omics data confirmed the core microorganism in millet fermentation, and further suggested *L. harbinensis* can promote the release and metabolism of specific phenolic compounds (Yan et al., 2025). Similarly, using multi-omics data, the correlations between the microbial community and aroma substances in millet *Huangjiu* have been established [64]. However, the traditional single-variable correlation analysis methods showed high false positive rates when analyzing multi-omics data (Weiss et al., 2016). Using the neural-network-based analysis method, the accuracy of predictions can be effectively improved (Morton et al., 2019). Besides, traditional sequencing techniques can only indicate the overall metabolic function of the microbial community. With the help of metagenome-assembled genomes, it is possible to effectively correlate the composition of the microbial community with its metabolic functions (Almeida and De Martinis, 2021). These emerging analytical methods will help to further explore the microbial metabolic mechanisms in millet fermentation.

### 7.2 Artificial intelligence-powered techniques

The fermentation process of millet is influenced by multiple factors such as the batch of raw materials, environmental factors, and fermentation parameters (Pan et al., 2020; Lu et al., 2024). It is difficult to effectively guide the subsequent fermentation process based solely on the data from a single fermentation batch. Conducting in-depth analysis of high-dimensional data from multiple batches can effectively guide the fermentation process. For high-dimensional data, traditional empirical methods always fail to capture non-linear interactions and temporal dependencies (Khan et al., 2022; Wang et al., 2025b). The application of artificial intelligence and machine learning techniques will offer a transformative solution. Artificial neural network (ANN) is a data processing system, which can simulate and compute the implicit linkages in the fermentation process (Alvarado et al., 2025; Shomope et al., 2025). ANN have been considered as an effective way to predict biological processes. Ramirez et al. created an ANN with input parameters of total solids, volatile solids, lipids, and proteins, which can effectively predict methane production (Dominguillo-Ramírez et al., 2023). Combining response surface analysis with ANN, the fermentation process can be effectively optimized. Based on the combined utilization of ANN and response surface analysis, the yield of chlorophyll that produced by an engineering consortium was increased by 2.1 times (Guendouzi et al., 2024). With the help of artificial intelligence-powered machine learning models, researches can reconstruct GSMM and further predict the physiological traits of certain species. For example, an artificial intelligence-enhanced GSMM for *Bacillus subtilis* was established by Bi et al. This model reached an 87.9 % accuracy in predicting gene expression and metabolites synthesis under various conditions (Bi et al., 2024). However, these artificial intelligence-powered tools need high-quality datasets, which sometimes is difficult to obtain due to cost and detection accuracy limitations. Subsequent research should focus on improving data quality in order to obtain more accurate guidance.

### 7.3 Online detection of fermentation process

The fermentation process exerts significant impacts on the safety, stability and quality formation of the fermented product. Traditional fermentation monitoring relies on intermittent sampling and offline analysis (Yamauchi et al., 2026). However, these methods may lead to the lag of data acquisition and microbial contamination. With the continuous development of sensor technology, spectral imaging technology and machine learning technology, online monitoring of key indicators in the cereal fermentation process is constantly receiving attention (Zhou et al., 2018; Simoska et al., 2021). In the field of fermentation, spectroscopy-based techniques are the most commonly used online detection method. With the help of Fourier transform infrared spectroscopy (FTIR), Raman spectroscopy, near-infrared spectroscopy (NIR) and fluorescence spectroscopy, compositional and structural changes of fermentation material can be assessed instantaneously (Ding et al., 2025). Deep learning models were applied to spectral data by Deng et al. to quantitatively detect mycotoxins in cereals. Through the application of transfer learning strategy, FTIR spectrometry achieved an R^2^ of 0.9356, a relative prediction deviation (RPD) of 3.9497 for wheat zearale none prediction (Deng et al., 2025). In another study, NIR was combined with partial least squares regression analysis, and this method was used to predict the solid-state fermentation stage of vinegar. This method achieved a 100% accuracy ratio of solid-state fermentation stage judgment (Fan et al., 2020). Compared with spectral technology, colorimetric sensor-based method is considered as a cost-effective option. Wang et al. colorimetric sensor to quantitative detect the total acids during apple vinegar fermentation. The correlation coefficient, root mean square error of prediction and RPD of the quantitative model were 0.8708, 0.0545 and 10.91, respectively (Wang et al., 2022a). However, the accuracy of the data obtained from a single type of sensor still needs to be improved (Jiang et al., 2022). Further research could attempt to conduct collaborative modeling of multiple sensor data, thereby enhancing the robustness of the data.

## 8 Conclusion and future prospects

This review highlights the role of fermentation in producing healthy millet product. Compared with traditional spontaneous fermentation, the inoculation of single strain or SynCom exhibits higher efficiency and stronger stability, and is gradually becoming the mainstream industrial production pattern. Parameters such as temperature, time, pH and inoculation volume were considered as key factors that affect the microbial succession, the nutritional profile and the efficiency of millet fermentation. Utilization of millet nutrients, such as the digestive characteristics of starch, and the bioavailability of polyphenols and minerals can be modified via fermentation. The application of emerging technologies such as omics integration strategies, artificial intelligence models and online detection techniques can contribute to further exploring the fermentation mechanism and enhancing the fermentation efficiency and stability. The current review provides new insights into the bio-utilization of nutrients in fermented millet, and also clarifies the great potential of emerging technologies for the fermentation of millet.

Despite these advances, several key challenges still remain to be resolved. For example, the microbial strain resources suitable for the fermentation of millet still need to be further exploited. The biological safety and metabolic potential of these strains still require further analysis. High-throughput screening technology, as well as whole-genome sequencing of microbial strains, can provide solutions for the aforementioned requirements. Besides, the interaction mechanisms among microorganisms during the fermentation process of millet and their impact on the quality of the product are still not clear. Subsequent studies can predict the potential interactions among microorganisms using the GSMM model, and further validate the key interaction relationships through wet experiments. Moreover, the current omics-based studies often exist the problems such as insufficient sample size and low sequencing depth, which consequently affect the accuracy of the analysis results. Future studies are recommended to adopt high-resolution detection techniques and conduct strict quality control over the experimental data before analysis.
